# Advancing genotoxicity assessment with transcriptomic biomarkers: from 2D to 3D HepaRG spheroid models

**DOI:** 10.1093/toxsci/kfag095

**Published:** 2026-08-22

**Authors:** Mariam Saleh, Kevin Hogeveen, Anouck Thienpont, Emmanuel Demuynck, Valérie Fessard, Birgit Mertens, Ludovic Le Hégarat

**Affiliations:** Toxicology of Contaminants Unit, Fougères Laboratory, French Agency for Food, Environmental and Occupational Health and Safety (ANSES), Fougères, 35306, France; Toxicology of Contaminants Unit, Fougères Laboratory, French Agency for Food, Environmental and Occupational Health and Safety (ANSES), Fougères, 35306, France; Department of Chemical and Physical Health Risks, Sciensano, Brussels, 1050, Belgium; Department of Chemical and Physical Health Risks, Sciensano, Brussels, 1050, Belgium; Department of In Vitro Toxicology and Dermato-Cosmetology, Vrije Universiteit Brussel, Brussels, 1090, Belgium; Toxicology of Contaminants Unit, Fougères Laboratory, French Agency for Food, Environmental and Occupational Health and Safety (ANSES), Fougères, 35306, France; Department of Chemical and Physical Health Risks, Sciensano, Brussels, 1050, Belgium; Toxicology of Contaminants Unit, Fougères Laboratory, French Agency for Food, Environmental and Occupational Health and Safety (ANSES), Fougères, 35306, France

**Keywords:** new approach methodologies (NAMs), TGx-DDI, GENOMARK, genotoxic (GTx), non-genotoxic (NGTx), non-genotoxic carcinogens (NGTxC)

## Abstract

The development of New Approach Methodologies (NAMs) for genotoxicity testing is needed to support the transition toward animal-free human-relevant chemical genotoxicity assessment. In this context, we investigated the predictive power of two transcriptomic biomarkers developed in 2D cell systems (GENOMARK and TGx-DDI) in metabolically active 3D HepaRG spheroids to classify chemicals as genotoxic (GTx) or non-genotoxic (NGTx). Human-hepatic HepaRG spheroids were treated for 72 h with three different groups of chemicals: (i) five genotoxic chemicals: Lasiocarpine, cyclophosphamide, ethyl methanesulfonate, aflatoxin B1, benzo[a]pyrene; (ii) five non-genotoxic non-carcinogens: Aflatoxin B2, 2-deoxy-D-glucose, D-mannitol, caffeine, ampicillin trihydrate; and (iii) five non-genotoxic carcinogens: Perfluorooctanoic acid, diethanolamine, thioacetamide, phorbol-12-myristate-13-acetate, di(2-ethylhexyl) phthalate. Gene expression data from the treated HepaRG spheroids were collected using the high-throughput TempO-Seq technology and analyzed with the transcriptomic biomarkers GENOMARK and TGx-DDI. Results with both biomarkers generally aligned with existing knowledge for all three groups of tested chemicals, effectively distinguishing genotoxic from non-genotoxic chemicals. The results of this study indicate that combining the 3D HepaRG spheroid model with transcriptomic biomarkers offers a promising, human-relevant approach to complement traditional *in vitro* genotoxicity tests.

## Graphical Abstract

**Figure:**
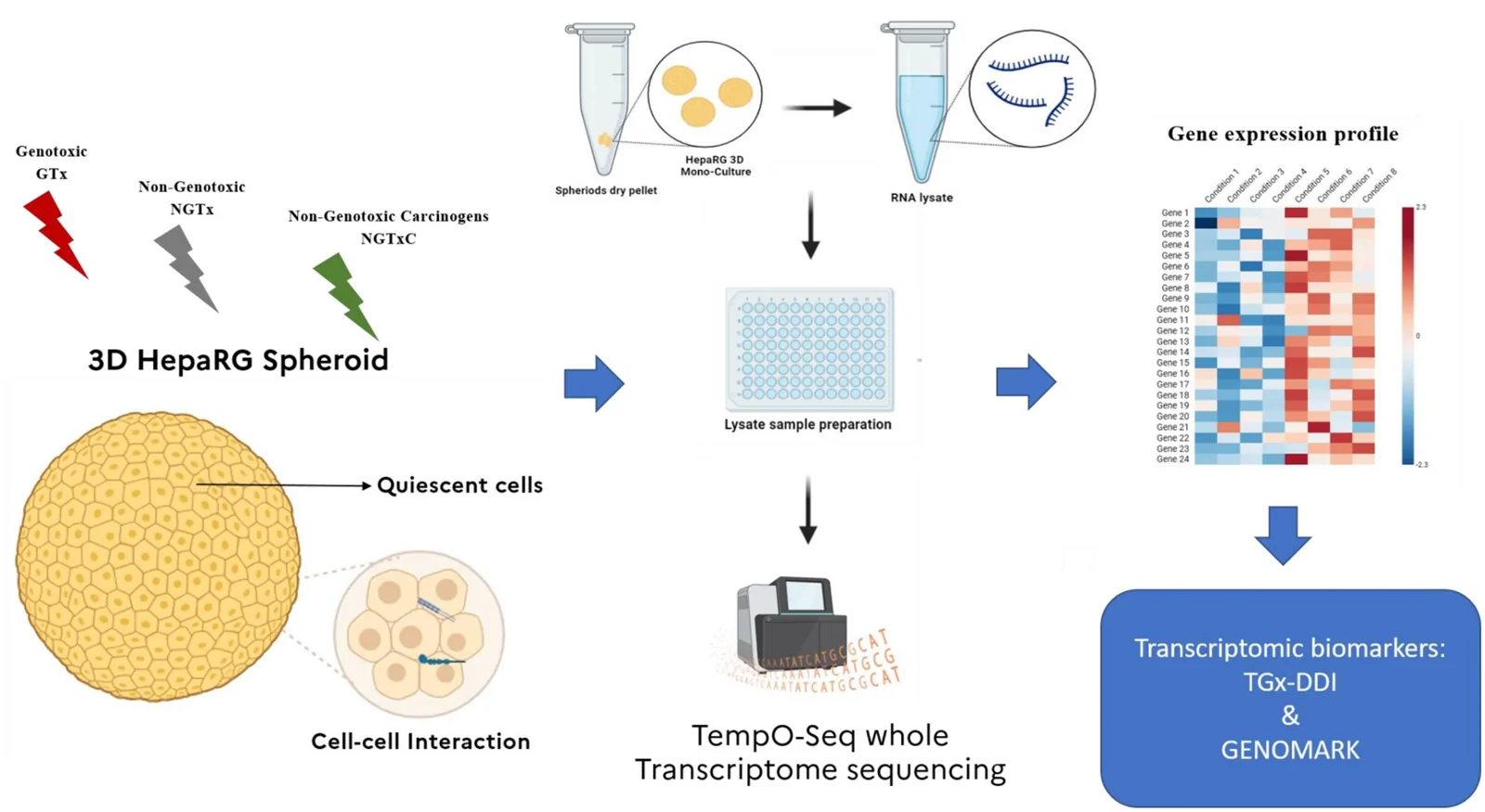

Genotoxicity assessment is a critical element in Chemical Safety Assessment (CSA) (Turkez et al. 2017; Sachse et al. 2026). Currently, *in vitro* testing batteries are available to evaluate the genotoxic potential of chemicals (Cartus and Schrenk 2017). These testing batteries are designed to identify both gene mutations and chromosomal alterations. Gene mutations are assessed with assays such as the Ames test, and mammalian gene mutation assays (Chen et al. 2022b). Chromosomal alterations are typically detected using the *in vitro* chromosome aberration assay or the *in vitro* micronucleus (MN) assay (Zeller et al. 2018). However, in most regulatory settings, positive *in vitro* results need to be followed up with an adequate *in vivo* test to confirm the *in vitro g*enotoxic effect (Nesslany 2017; Fortin et al. 2023). Consequently, most current genotoxicity testing strategies still rely on animal testing.

Thus, significant efforts are ongoing to develop New Approach Methodologies (NAMs) that can reduce the need for *in vivo* testing (Luijten et al. 2021; Fortin et al. 2023; Menz et al. 2023). An example includes the high-throughput transcriptomic approaches that are being integrated into NAMs to evaluate the genotoxic potential of chemicals (Buick et al. 2021). To facilitate the analysis of large transcriptomics datasets, selected sets of biomarker genes for genotoxicity have been identified in human-relevant cell lines (Li et al. 2025). By analyzing the chemical-induced expression profiles of these genes using machine learning methods, these biomarkers allow the evaluation of DNA damage caused by different chemicals (Li et al. 2025).

In 2022, the International Workshops on Genotoxicity Testing (IWGT) convened a workshop to assess advancements in the development and application of transcriptomic biomarkers for genotoxicity testing. The working group identified several promising biomarker candidates. Among others, TGx-DDI and GENOMARK are the most widely applied in genotoxicity testing (Li et al. 2025). Furthermore, Health Canada’s GeneTox21 program, proposed the integration of TGx-DDI biomarker into the current genotoxicity hazard assessments (Fortin et al. 2023).

The TGx-DDI biomarker consists of a 64-gene set derived from TK-6 cells exposed to different genotoxic substances for a short exposure time (3 h) (Barutcu 2024). The TGx-DDI gene set signature has demonstrated strong reliability in distinguishing genotoxic agents from non-genotoxic agents (Allemang et al. 2021; Chen et al. 2022a) using a variety of different cell lines (including HepaRG cells) and gene expression technologies (Cho et al. 2019; Buick et al. 2020, 2021, 2022). Similarly, the GENOMARK transcriptomic biomarker includes 84 genes selected from human 2D HepaRG cells exposed to various compounds, including genotoxic and non-genotoxic compounds, for a longer exposure time (72 h) (Thienpont et al. 2024). GENOMARK has also shown good predictivity and accuracy in distinguishing genotoxic from non-genotoxic substances (Ates et al. 2018; Thienpont et al. 2023). Interestingly, Thienpont et al. (2024) demonstrated that combining both the TGx-DDI and GENOMARK biomarkers on transcriptomic data generated by the high-throughput TempO-Seq sequencing technology in 2D HepaRG cells can improve chemical genotoxicity assessment (Thienpont et al. 2024).

TGx-DDI has been validated for the classification of DNA damage-inducing chemicals in several *in vitro* systems; however, its application in 3D liver models remains limited. Most studies have relied on conventional 2D cell systems, particularly TK6 cells and HepaRG cells. Only a few studies have explored the use of TGx-DDI in 3D liver models. For example, TGx-DDI analysis has been applied to primary human hepatocytes (PHH) exposed to per- and polyfluoroalkyl substances, demonstrating the potential of transcriptomic biomarkers to evaluate genotoxic mechanisms in complex liver systems (Rowan-Carroll et al. 2025). In addition, the GENOMARK transcriptomic biomarker has been predominantly implemented in 2D *in vitro* platforms, with no reported evaluation in 3D liver systems. Overall, the broader applicability of transcriptomic biomarkers in 3D hepatic models remains underexplored. Therefore, expanding the application of these transcriptomic approaches to advanced 3D hepatic models may enhance their physiological relevance and improve the mechanistic understanding of chemical-induced genotoxicity.

In recent years, the development of 3D HepaRG spheroid model has gained momentum. Human hepatic HepaRG cells are of specific interest for genotoxicity testing because, compared with other hepatic cell lines, they demonstrate enhanced physiological and metabolic competence. They are characterized by a high expression of key Cytochrome P450 enzymes, functional activation of nuclear receptors, and a bipotent differentiation capacity that allows the formation of both hepatocyte- and cholangiocyte-like cells (Anthérieu et al. 2012; Ranade et al. 2014; Guo et al. 2024a). Although 3D cultures tend to be associated with higher costs and increased technical complexity, they offer several advantages over traditional 2D monolayers. Notably, 3D HepaRG spheroids display improved differentiation stability, enhanced metabolic functionality, and a cellular architecture that is more physiologically relevant (Rabiet et al. 2024).

Their suitability for high-throughput genotoxicity testing has also been demonstrated. For example, in the CometChip assay, 3D HepaRG spheroids showed greater sensitivity to genotoxicants, detecting effects at lower benchmark doses than 2D monolayers (Barranger and Le Hégarat 2022). Similarly, in a high-throughput flow cytometry-based micronucleus assay, 3D HepaRG spheroids outperformed 2D cultures in identifying indirect-acting genotoxicants and carcinogens requiring metabolic activation (Seo et al. 2023). When compared with other hepatic 3D models, such as HepG2 spheroids or primary human PHH, 3D HepaRG spheroids also demonstrate greater adaptability to high-throughput platforms (Quintás et al. 2021; Guo et al. 2024b).

In this study, we applied an in vitro high-throughput transcriptomic methodology to investigate the applicability and predictive power of the two transcriptomic biomarkers (i.e. GENOMARK and TGx-DDI) for detecting the genotoxic potential of chemicals in 3D HepaRG model.

## Materials and methods

### HepaRG cell culture

The human hepatic HepaRG cell line was purchased from Biopredic International (Rennes, France) and cultured as previously described (Saleh et al. 2026). Briefly, HepaRG cells were cultured in Williams E medium 1X + Glutamax-I (Gibco, Noisy le Grand, France) supplemented with 10% FBS FetalClone II (Cytiva, Saint Germain en Laye, France), Penicillin 5,000 units/mL/Streptomycin 5,000 µg/mL (Gibco, Noisy le Grand, France), 5 µg/mL insulin (Sigma, Saint Quentin Fallavier, France), and 18 µg/mL Hydrocortisone hemisuccinate (Upjohn, Paris, France). HepaRG cells between passages 15 to 18 were seeded in 75cm^2^ flasks (VWR, Rosny sous Bois, France) at a density of 20 × 10^3^ cells/cm^2^ and incubated at 37 °C with 5% CO_2_. Cell dissociation with TryplE 1X (Thermo Fisher Scientific, Waltham, United States) was performed after 2 wk in 75cm^2^ flasks at a density of 27 × 10^3^ cells/cm^2^ cells to start differentiation with DMSO (Sigma, Saint Quentin Fallavier, France).

### HepaRG in a 3D culture system

As previously described by Saleh et al. (2026), after 14 d of differentiation in 1.7% DMSO (Fig. 1), cells were trypsinized, and seeded in a 96-well ultra-low attachment (ULA) Plates (Sigma, Saint Quentin Fallavier, France) at a density of 2,000 cells/well. Fresh media without DMSO was replaced every 2 d. Spheroids were ready for chemical exposure 1 wk after seeding (Saleh et al. 2026).

**Fig. 1.**
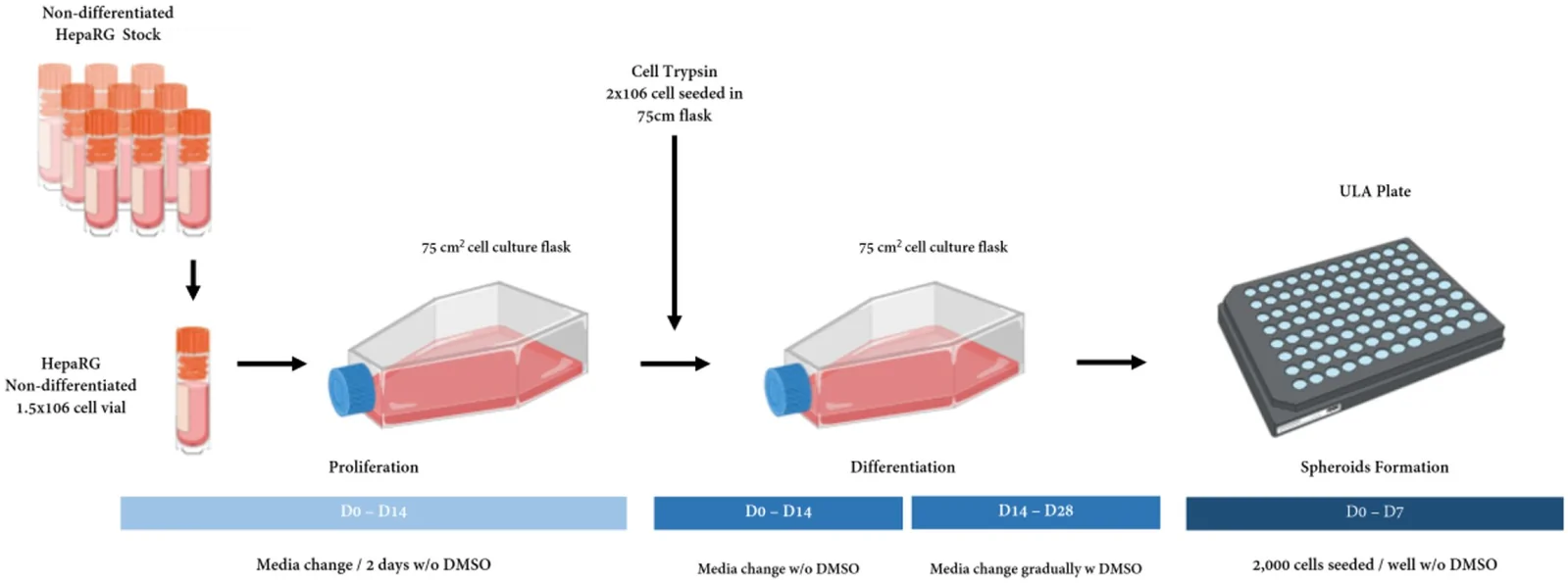
3D HepaRG spheroid culture protocol (Saleh et al. 2026).

### Chemical selection

Fifteen chemicals were selected in this study (Table 1). Five known Genotoxic (GTx) chemicals: Lasiocarpine (PA), cyclophosphamide (CPA), ethyl methanesulfonate (EMS), aflatoxin B1 (AFB1), and benzo[a]pyrene (B[a]P); five Non-Genotoxic (non-GTx) chemicals: Aflatoxin B2 (AFB2) 2-deoxy-D-glucose (2DDG), D-mannitol, caffeine, and ampicillin trihydrate; and five Non-Genotoxic Carcinogens (NGTxC): Perfluorooctanoic acid (PFOA), diethanolamine (DEA), thioacetamide (TAA), phorbol-12-myristate-13-acetate (TPA), and di(2-ethylhexyl) phthalate (DEHP)). All chemical solutions were prepared in 1% DMSO (Sigma, Saint Quentin Fallavier, France). Chemical treatments were performed in serum-free media. All chemicals were purchased from Sigma (Saint-Quentin-Fallavier, France) except PA, which was from Phytoplant Research S.L. (Córdoba, Spain).

**Table 1.** Chemicals selected for this study.

Chemicals	CAS	MoA	*In vitro*		*In vivo*	Genomark/TGx-DDI 2D HepaRG	Concentrations (µM)		
			Ames	MN			IC_10_	IC_10/2_	Δ
**GTx**									
Ethyl methanesulfonate (EMS)	62-50-0	Hepatotoxic Alkylation	**+** (Lim et al. 2025)	**+** (Lim et al. 2025)	+ Rodent Boiassay (Zhu et al. 2022)	**+** (Thienpont et al. 2024)	1,000	500	N/A
Aflatoxin B1 (AFB1)	1162-65-8	Hepatotoxic DNA Adducts	**+** (Wong and Hsieh 1976)	**+** (Marchese et al. 2018)	+ in vivo micronucleus and comet assay in Rats (Corcuera et al. 2015)	**+** (Thienpont et al. 2024)	0.1	0.05	N/A
Benzo[a]pyrene (BaP)	50-32-8	Hepatotoxic DNA adducts	**+** (Phillipson and Ioannides 1989)	**+** (Lim et al. 2025)	**+** in vivo micronucleus in mouse (Long et al. 2016)	**+** (Buick et al. 2021) *Unpublished data + GENOMARK	3.6	1.8	5
Cyclophosphamide (CPA)	6055-19-2	Hepatotoxic Alkylation	**+** (Anderson et al.1995)	**+** (Anderson et al. 1995) (Yadav et al. 2023)	**+** in vivo micronucleus in mouse (Krishna et al. 1995)	**+** (Thienpont et al. 2024)	2,000	1,000	N/A
Lasiocarpine (PA)	303-34-4	Hepatotoxic Alkylation	**+** (Yamanaka et al. 1979)	**+** (Li et al. 2020)	**+** in vivo rat (Xia et al. 2006)	ND	0.5	0.25	N/A
**Non-GTx**									
Caffeine (CAFF)	58-08-2	Not hepatotoxic	–	–	–	ND	5,000	2,000	N/A
Ampicillin trihydrate (AMP)	7177-48-2	Not hepatotoxic	–	–	–	– (Buick et al. 2020; Thienpont et al. 2023)	10,000	N/A	N/A
Aflatoxin B2 (AFB2)	7220-81-7	Unknown Hepatotoxicity	ND	ND	ND	ND	15	7.5	10
2-Deoxy-D-glucose (2DDG)	154-17-6	Not hepatotoxic	–	–	–	– (Buick et al. 2020; Thienpont et al. 2023)	10,000	N/A	N/A
D-mannitol (D-man)	69-65-8	Not hepatotoxic	–	–	–	ND	10,000	N/A	N/A
**NGTxC**									
Di(2-ethylhexyl) phthalate (DEHP)	117-81-7	Hepatotoxic PPARα nuclear receptor activation	–	–	–	ND	200	100	N/A
Thioacetamide (TAA)	62-55-5	Hepatotoxic Oxidative stress	–	–	–	ND	7,000	3,500	N/A
Diethanolamine (DEA)	111-42-2	Hepatotoxic Oxidative stress Phospholipid metabolism	–	–	–	ND	200	100	N/A
Perfluorooctanoic acid (PFOA)	3825-26-1	Hepatotoxic Oxidative stress Mitochondrial activity	–	–	–	ND	40	20	N/A
*12-O-Tetradecanoylphorbol-13-acetate* (TPA)	16561-29-8	Hepatotoxic Promoting cell proliferation	–	–	–	ND	20	10	5

The table presents the full name of the chemicals and the abbreviation, the CAS number, the Mode of Action (MoA), previously published *in vitro* genotoxicity information collected with the Ames test and the micronucleus test (MN), previously published *in vivo* genotoxicity information, previously published data obtained with GENOMARK/TGx-DDI in the 2D HepaRG cell system and the concentrations tested in this study. (Δ), additional tested concentrations; (ND), no data; (N/A), not applicable, (*) Unpublished data.

### Cell viability assay

A pre-cytotoxicity assay was performed for all the selected chemicals to determine the non-toxic concentrations for the transcriptomic experiments to allow a stable, low-stress transcriptional response where DNA-damage signaling can be detected without the activation of stress pathways, to avoid false positive responses. For the GTx and NGTxC chemicals, six concentrations were selected for the pre-cytotoxicity assay based on a previously published toxicity range in 3D HepaRG spheroids with the same assay. For the non-GTxschemicals, the six different concentrations were selected starting from the highest soluble concentrations (not more than 10 mM) (SF1, SF2, SF3). After a 72 h repeated dose treatment, cytotoxicity was assessed using the CellTiter-Glo 3D Cell Viability kit (Promega, Charbonnières-les-Bains, France), according to the manufacturer’s instructions. One spheroid per condition was used for the cytotoxicity measurements. ATP luminescence was measured using a FLUOstar Optima microplate reader (BMG Labtech, Champigny-sur-Marne, France). Blank-corrected raw data were used to calculate the relative cell viability using the following formula:


Relative ATP level (%)=[Blank corrected raw data (sample) /Blank corrected raw data (control)] × 100

Next, IC_10_, IC_50_, and IC_90_ values of each chemical were calculated based on the relative ATP values using GraphPad prism (version 9.5.1). In addition, concurrent cytotoxicity assays were conducted with the selected IC_10_ and IC_10/2_ (half the IC_10_ concentration), as described above, to assess cytotoxicity across all conditions tested in the transcriptomics study (Table 1).

### Chemical exposure

Based on the results of the pre-cytotoxicity tests, concentrations equivalent to the IC_10_ and IC_10/2_ were selected, except for 3 out of the 5 non-GTx chemicals (2DDG, MAN, and D-MAN) for which only a single concentration equivalent to the IC_10_ was tested. Moreover, an additional concentration was tested for B[a]P and AFB2 as the pre-cytotoxicity results differed from previously collected in-house or literature data, resulting in a total of 3 concentrations for these two compounds (Table 1). Moreover, since the IC_50_ of TPA was close to IC_10_ (SF3), an additional concentration (5 µM) was also included in this study.

Spheroids were treated with the selected concentrations of the chemicals for 72 h, with media being replaced every 24 h. After exposure (Table 1), 32treated spheroids per condition were collected in an eppendorf tube and centrifuged for 3 min at 500 × *g*. After washing twice with PBS, spheroids were stored as a dry pellet at −80 °C before RNA-lysate preparation. As previously mentioned, concurrent cytotoxicity measurements were performed for one spheroid/tested concentration. Moreover, this experimental design was repeated independently 3 times using different HepaRG cell batches.

### RNA lysate preparation

BioSpyder Enhanced 2X Lysis Buffer (P/N 201247) was diluted with PBS and used as a 1X solution to lyse 32 spheroids/condition and prepare the RNA-isolated lysate following the TempO-Seq Assay user guide. In brief, spheroids were incubated for 10 min with the lysis buffer at 37 °C. The lysate samples were shipped on dry ice to Bioclavis (Glasgow, United Kingdom), a spinoff company of BioSpyder Technologies.

### TempO-Seq assay

Gene expression profiles of the spheroids treated with the controls or chemicals were determined using the high-throughput TempO-Seq technology. The Human whole transcriptome (v2.1) was used as probe set along with the TempO-seq Human whole transcriptome reagent kit to prepare libraries in 96-well plate format. One negative control (1X TempO-seq Lysis buffer only) and two positive qPCR assay controls (Human Reference Total RNA) (Brain total RNA) were used as four replicates in the analysis. Briefly, RNA-lysate samples were hybridized under a temperature gradient to the whole transcriptome probe pool through the complementary sequence between mRNA and primer binding sites. After hybridization, the unbound probes were removed through nuclease digestion. The amplification templates of the bound probes were then amplified using a primer set (single-plex PCR reaction, with a single primer pair for each sample) PCR reaction to add sample-specific tags and sequencing adapters. The amplified libraries were then sequenced using an Illumina NextSeq 2000 system, yielding a median read depth of 6 million reads per sample. This TempO-Seq sequencing protocol ensures high specificity and sensitivity as described in detail by Yeakley et al. (2017).

### Transcriptomic biomarker-based chemical classification

The gene expression profiles of the tested chemicals were analyzed using the bioinformatics tools of the GENOMARK and TGx-DDI biomarkers as described previously in Thienpont et al. (2024). In brief, gene expression data generated by the TempO-Seq technology were transformed into log2 values. For both biomarkers, genes having multiple probe IDs were summed prior to the log transformation. For GENOMARK, the support vector machine (SVM) using R packages e1071 and caret, and the Random Forest (RF) algorithm using RF package in R (version 4.0.4) were used to compute prediction scores. The prediction scores are values between 0 and 1, and can lead to three different predictions: Positive if the score is > 0.55, negative if the score is < 0.45, and equivocal if the score is between 0.55 and 0.45. When both algorithms returned a score > 0.55, the samples are classified as genotoxic. When both algorithms returned a score < 0.45, the sample is classified as non-genotoxic. If one of the two prediction scores is between 0.45 and 0.55, the prediction is set as inconclusive for the sample. Finally, if the two methods returned a different prediction, the sample is also considered as Inconclusive. For TGx-DDI, the Nearest Shrunken Centroids (NSCs) method, implemented in the TGx-DDI classification tool (TGx-DDI Biomarker for DNA Damage Classification), was conducted to classify the samples. The method returns two NSC probabilities; a DNA Damage Inducing (DDI) probability and a Non-DNA Damage Inducing probability (NDDI). The sample was classified as DDI when the NSC analysis yielded a higher DDI probability than the NDDI probability. In the opposite case, the sample was classified as NDDI.

## Results

### Cytotoxicity

Pre-cytotoxicity evaluations were performed for all the selected chemicals to determine the concentrations corresponding to the IC_10_ and IC_10/2_ (SF1, SF2, SF3). In addition, concurrent cytotoxicity evaluations were conducted together with the transcriptomics study to ensure that no severe cytotoxic effects were present. The results of the former showed that all treatment conditions tested with 3D HepaRG spheroids for a 72 h repetitive treatment exhibited no more than 30% associated cytotoxicity, except for the highest concentrations tested with B[a]P (10 µM) and TPA (20 µM) (Fig. 2).

**Fig. 2.**
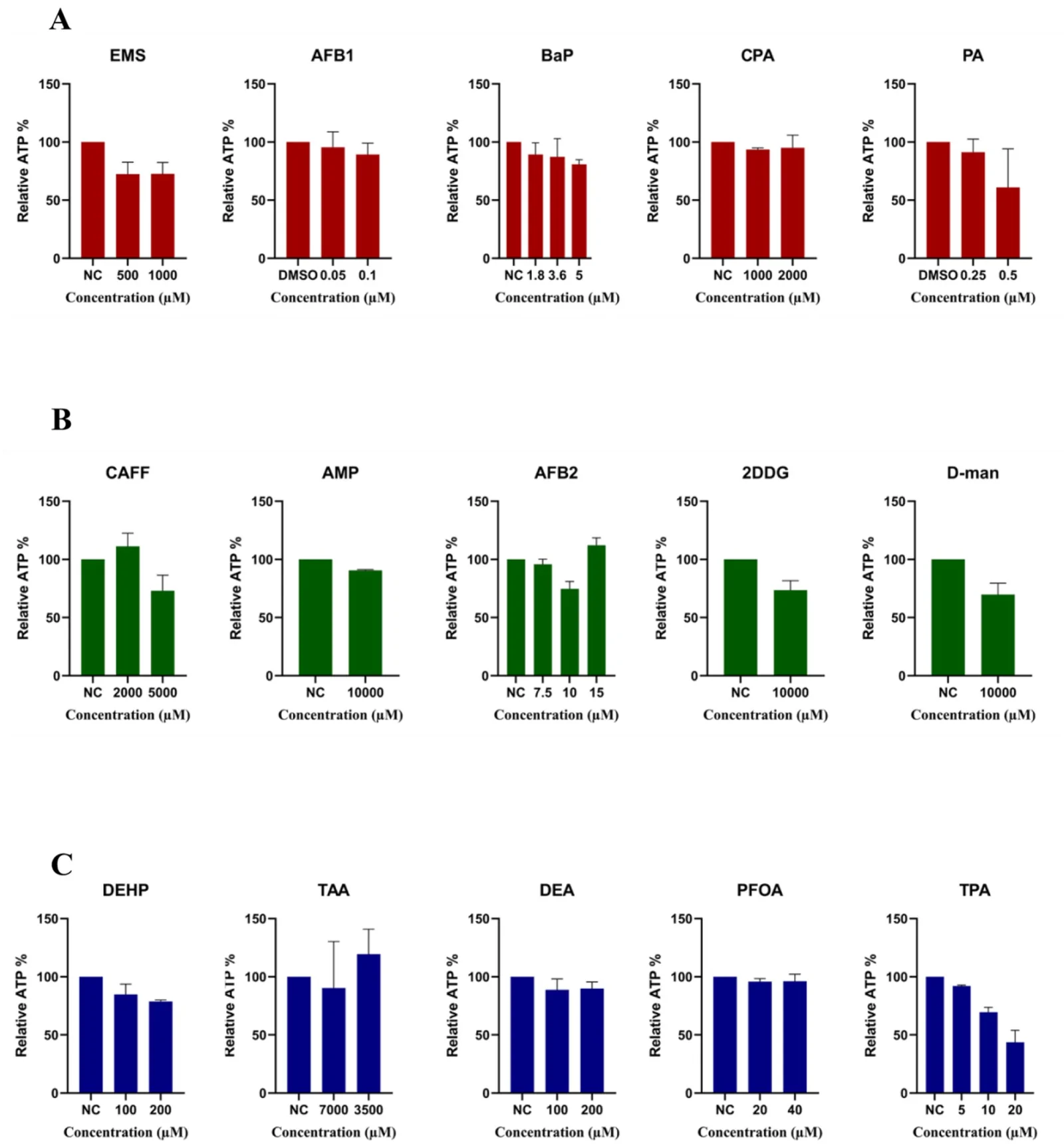
Concurrent cytotoxicity assay to verify the selected concentrations of the principal study. Relative ATP levels following 72 h repetitive treatments with the selected concentrations of each chemical in 3D HepaRG spheroids. (A) Genotoxic chemicals (GTx): EMS, AFB1, BaP, CPA, and PA. (B) Non-genotoxic chemicals (non-GTx): CAFF, AMP, AFB2, 2DDG, and D-man. (C) Non-Genotoxic Carcinogens (NGTxC) chemicals: DEHP, TAA, DEA, PFOA, and TPA. Data are presented as means ± SEM of 3 independent biological replicates.

### Transcriptomic biomarker-based classification results

A result is considered as positive with TGx-DDI if it is classified as DDI in the statistical prediction model used. The TGx-DDI transcriptomic biomarker correctly classified all GTx chemicals as DDI chemicals (Table 2). EMS and B[a]P were classified as DDI only at the highest concentration tested. Among the non-GTxchemicals, only AFB2 was classified as DDI. In addition, the highest concentration tested for CAFF (5,000 µM), also resulted in a classification as DDI with the TGx-DDI biomarker. Moreover, all chemicals in the NGTxC group were classified as non-DDI, with the exception of TPA, which was classified as DDI at a cytotoxic concentration close to the IC_50_ of 20 µM (Table 2).

**Table 2.** Overview of the TGx-DDI and GENOMARK classification results based on gene expression profiles from 3D HepaRG spheroids obtained with TempO-Seq and analyzed with the different statistical tools.

Biomarker			GENOMARK			TGx-DDI	
Chemical	Condition	Concentration µM	RF_GTX	SVM_GTX	Prediction	DDI_probability	Prediction
**GTx**							
EMS	C1	1000	0,58	0,75	POSITIVE	1	DDI
	C2	500	0,33	0,21	NEGATIVE	0,0000196	NDDI
CPA	C1	2000	0,85	0,98	POSITIVE	1	DDI
	C2	1000	0,56	0,55	POSITIVE	1	DDI
AFB1	C1	0,1	0,61	0,82	POSITIVE	1	DDI
	C2	0,05	0,45	0,23	INCONCLUSIVE	1	DDI
B[a]P	C1	5	0,94	0,99	POSITIVE	1	DDI
	C2	3,6	0,37	0,09	NEGATIVE	8,66E-29	NDDI
	C3	1,8	0,28	0,02	NEGATIVE	5,05E-34	NDDI
PA	C1	0,5	0,84	0,98	POSITIVE	1	DDI
	C2	0,25	0,62	0,62	POSITIVE	1	DDI
**non-GTxs**							
MAN	C1	10000	0,40	0,11	NEGATIVE	1,78E-56	NDDI
CAFF	C1	5000	0,66	0,45	INCONCLUSIVE	1	DDI
	C2	2000	0,48	0,03	INCONCLUSIVE	1,01E-32	NDDI
AMP	C1	10000	0,47	0,30	INCONCLUSIVE	8,94E-36	NDDI
AFB2	C1	15	0,72	0,74	POSITIVE	1	DDI
	C2	10	0,55	0,31	INCONCLUSIVE	1	DDI
	C3	7,5	0,59	0,26	INCONCLUSIVE	1	DDI
2DDG	C1	10000	0,48	0,48	INCONCLUSIVE	3,38E-36	NDDI
**NGTxCs**							
PFOA	C1	40	0,35	0,12	NEGATIVE	4,5E-59	NDDI
	C2	20	0,29	0,08	NEGATIVE	1,99E-60	NDDI
DEA	C1	200	0,50	0,31	INCONCLUSIVE	1,64E-59	NDDI
	C2	100	0,41	0,22	NEGATIVE	1,27E-35	NDDI
DEHP	C1	200	0,41	0,55	INCONCLUSIVE	3,89E-37	NDDI
	C2	100	0,30	0,05	NEGATIVE	3,75E-32	NDDI
TAA	C1	7000	0,44	0,10	NEGATIVE	1,66E-34	NDDI
	C2	3500	0,25	0,04	NEGATIVE	2,54E-43	NDDI
TPA	C1	20*	0,72	0,57	POSITIVE*	1	DDI*
	C2	10	0,24	0,01	NEGATIVE	1E-79	NDDI
	C3	5	0,31	0,01	NEGATIVE	1,43E-78	NDDI

(DDI) represents the positive results, (NDDI) represents the negative results, and (*): represents toxic concentrations.

The GENOMARK biomarker classifies chemicals as either positive or negative when both statistical predictive models give similar prediction scores, i.e. two scores > 0.55 or two scores < 0.45, respectively. A sample is classified as inconclusive when there are different calls between the SVM and RF prediction models, or when at least one of the two models returns an equivocal prediction score (between 0.45 and 0.55). The GENOMARK biomarker correctly classified all GTx chemicals as positive (Table 2). As with results from TGx-DDI, EMS, and B[a]P were classified as DDI only at the highest concentration tested. Among the non-GTx chemicals, only AFB2 was classified as DDI. However, CAFF, 2DDG, and AMP (non-GTx) showed inconclusive results at the concentrations tested. NGTxC chemicals were classified negative with GENOMARK, with the exception of TPA at a toxic concentration near the IC_50_ (20 µM) (Table 2). Inconclusive results were obtained for the highest concentrations of DEHP and DEA.

### Overall classification with both biomarkers

Results of both biomarkers were combined to obtain an overall conclusion on the genotoxic potential of the tested chemicals. Chemicals were considered genotoxic if they were classified as inconclusive or positive by GENOMARK and as DDI by TGx-DDI. Chemicals were considered to be non-genotoxic if they were classified as negative or inconclusive by GENOMARK and as NDDI by TGx-DDI. If both biomarkers returned contradictory results (negative by GENOMARK and DDI by TGx-DDI, or positive by GENOMARK and NDDI by TGx-DDI), the chemical was considered equivocal. The classification of chemicals as GTx or NGTx with both biomarkers showed high accuracy, especially with the GTx and NGTxC group of chemicals (Table 3). Overall, the combined application of GENOMARK and TGx-DDI demonstrated strong predictive performance, correctly classifying 13 of the 15 chemicals tested (86.7%), supporting their complementary value.

**Table 3.** Summary of classification results with both applied biomarkers, TGx-DDI and GENOMARK.

Chemicals	GENOMARK	TGx-DDI	Overall conclusion
**GTx**			
EMS	Positive	DDI	GTx
AFB1	Positive	DDI	GTx
B[a]P	Positive	DDI	GTx
CPA	Positive	DDI	GTx
PA	Positive	DDI	GTx
**Non-GTxs**			
CAFF	INCONCLUSIVE	DDI	GTx
AMP	INCONCLUSIVE	NDDI	NGTx
AFB2	Positive	DDI	GTx
2DDG	INCONCLUSIVE	NDDI	NGTx
D-man	Negative	NDDI	NGTx
**NGTxCs**			
DEHP	INCONCLUSIVE	NDDI	NGTx
TAA	Negative	NDDI	NGTx
DEA	INCONCLUSIVE	NDDI	NGTx
PFOA	Negative	NDDI	NGTx
TPA	Negative	NDDI	NGTx

## Discussion

TGx-DDI and GENOMARK are gene set-based NAMs that aim to improve *in vitro* genotoxicity evaluation. They have been successfully applied, both qualitatively and quantitatively, to various gene expression datasets collected for GTx and NGTx chemicals in 2D HepaRG cells, one of the most widely used cell models in chemical hazard identification (Guo et al. 2024b). However, TGx-DDI has been evaluated in only a few physiologically relevant 3D liver models (e.g. PHH) (Rowan-Carroll et al. 2025), and its broader application in such models remains limited. GENOMARK, in contrast, has been used exclusively in 2D *in vitro* systems, with no reported implementation in 3D liver spheroids model. In this context, we conducted a qualitative *in vitro* genotoxicity test using both TGx-DDI and GENOMARK transcriptomic biomarkers in 3D HepaRG spheroids. Gene expression data were obtained using the TempO-Seq sequencing approach, for which its applicability with both biomarkers has previously been demonstrated (Thienpont et al. 2024).

All GTx chemicals tested in our study were correctly classified as DDI and GTx with TGx-DDI and GENOMARK, respectively. Although TGx-DDI and GENOMARK gene sets share only two genes in common (Bruin et al. 2003; Carvajal et al. 2012), they can both classify chemicals with known genotoxic effects with high accuracy using gene expression data collected in a 3D HepaRG test system. It should be noted that all the GTx chemicals (or their metabolites) tested in our study interact directly with the DNA. For instance, EMS is an alkylating agent that directly damages DNA by transferring ethyl groups to nucleophilic sites, leading to mutations (Pool et al. 1988; Jacociunas et al. 2012). In addition, CPA, AFB1, B[a]P, and PA damage DNA through their metabolites that strongly bind to the DNA and cause DNA and chromosomal damage (Chen and Liu 2019; Ebmeyer et al. 2019). Additional tests with, for example, aneugens are needed to assess how both biomarkers perform for compounds that do not directly interact with the DNA.

Both biomarkers classified B[a]P and EMS as genotoxic only at the highest concentration tested. This indicates that B[a]P at 1.6 and 1.8 µM, and EMS at 500 µM, may be too low to induce detectable genotoxic effects, highlighting the importance of testing at sufficiently high concentrations to initiate their genotoxic potential (Kopp et al. 2020). However, as with other genotoxicity assays in mammalian cells, care should be taken that the tested concentrations do not induce excessive cytotoxicity as such high toxicity levels may lead to non-specific effects.

For AFB1, an inconclusive result was obtained with the GENOMARK biomarker at 0.05 µM, whereas this concentration was already classified as DDI by the TGx-DDI biomarker. This finding is in line with the study by Thienpont et al. (2024), which reported that due to the underlying classification criteria, TGx-DDI generally classifies genotoxic agents as DDI at lower concentrations than GENOMARK (Thienpont et al. 2024; Li et al. 2025).

In contrast, several inconclusive results were obtained with the GENOMARK biomarker for NGTx chemicals that were classified as non-DDI by TGx-DDI. More specifically, CAFF, AMP, and 2DDG were classified as inconclusive with GENOMARK. However, it should be noted that these chemicals were tested at very high concentrations (i.e. CAFF at 2 and 5 mM and AMP and 2DDG both at 10 mM). Importantly, except for the highest concentration of CAFF, the probabilities of the two prediction models were always below 0.5 and the classification as inconclusive was due to a prediction score between 0.55 and 0.45 in one or two of the prediction models. Moreover, all concentrations of AMP and 2DDG were classified as NDDI by TGx-DDI. For this reason, the overall conclusion for AMP and 2DDG was non-genotoxic. In contrast, the highest concentration of CAFF was classified as DDI by TGx-DDI, indicating that at this concentration, some genes related to genotoxicity may be activated. Notably, in recent study, caffeine yielded negative results for genotoxicity at a maximum concentration of 100 µM following 24-h and 10-d treatments tested with 3D PPH spheroid (Rowan-Carroll et al. 2025).

Interestingly, AFB2 was classified overall as genotoxic, given that the highest concentration was positive in GENOMARK, and all concentrations were classified as DDI by TGx-DDI. AFB2 was included in the non-GTx group based on its negative *in vitro* genotoxicity in the γH2AX In-Cell Western assay in a study conducted by Theumer et al. (2018). However, some previous studies have shown that AFB2 binds to DNA and histones in rat liver *in vivo*, disturbing normal cellular processes (Groopman et al. 1981). Additionally, liver enzymes can metabolically activate AFB2, producing reactive intermediates, including AFB1. As shown in this study, AFB1 was classified as overall genotoxic and could possibly contribute to the genotoxic effects of AFB2 (Roebuck et al. 1978). The discrepancies between our findings and the previously reported negative results might be due to the cellular test system used. Indeed, for this study, 3D HepaRG spheroids were used, whereas most of the previous *in vitro* studies with AFB2 were performed in 2D cell systems (e.g. HepG2) (Theumer et al. 2018). It should also be noted that AFB1 exhibited genotoxicity at much lower concentrations (0.05 µM) compared with AFB2 (7.5 µM). Although unlikely, the positive results observed with AFB2 may be due to contamination of the AFB2 standard with AFB1. Given that the AFB2 standard has a reported purity of 99%, the remaining 1% impurity could contain a fraction of AFB1, potentially accounting for the positive genotoxicity result. Supplementary analyses, e.g. a differential gene expression analysis, could be applied to the dataset to obtain more insight into the relative expression of biomarker genes in samples leading to unexpected results such as for AFB2. Moreover, pathway analyses, such as Gene Set Enrichment Analysis (Subramanian et al. 2005),  could help to identify pathways leading to secondary genotoxicity.

Clear negative results for genotoxicity were obtained with the NGTxC chemicals with both biomarkers for most test conditions. Inconclusive results were obtained for the highest concentrations of DEA and DEHP. However, for both compounds, all probability scores were below (or equal to) 0.55 and the inconclusive result was due to a prediction score between 0.55 and 0.45 in one of the two GENOMARK prediction models. As all concentrations of both compounds were also classified as NDDI by TGx-DDI, they were concluded to be non-genotoxic. This is in agreement with the toxicological profiles of DEHP, TAA, DEA, PFOA, and TPA, as these compounds have been shown to be non-genotoxic, and to induce carcinogenicity through non-genotoxic modes of action. Indeed, they mainly exert their carcinogenic activity through epigenetic effects, oxidative stress, or receptor-mediated pathways, rather than by causing direct DNA damage (Veltman et al. 2023). It should be noted that the highest concentration of TPA tested in our study (20 µM) yielded a positive classification for genotoxicity with both biomarkers. This is probably due to cytotoxicity because considerable toxicity was observed in the ATP assay conducted in parallel with the principal study in 3D HepaRG cells. These findings highlight how GENOMARK and TGx-DDI can accurately identify true DNA damage-inducing agents from chemicals that cause cancer through non-genotoxic pathways in 3D systems.

Although TGx-DDI and GENOMARK have never been tested with 3D HepaRG spheroids, they demonstrated high classification accuracy when using 2D HepaRG cells. In a study conducted by Thienpont et al. (2024) the authors showed positive calls for AFB1, and CPA tested with 2D HepaRG cells. Moreover, Rowan-Carroll et al. (2025) reported that TGx-DDI classified AFB1, and CPA as positive genotoxic chemicals in 3D PHH, whereas PFOA was categorized as non-DDI after both short 24-h and longer 10-d treatments (Rowan-Carroll et al. 2025). These results are in line with our findings in a 3D HepaRG model, confirming the capability and applicability of both biomarkers on the gene expression profiles generated from 3D HepaRG spheroids treated with different GTx chemicals and NGTxC such as PFOA.

Despite the promising outcomes of our study, some limitations of this approach must be acknowledged. Although 3D HepaRG spheroids can be combined with high-throughput analysis/scoring technologies, the ULA culture system used in our study to conduct spheroid formation and chemical treatment suffers from low throughput, considering the number of spheroids required per treatment condition. Future work should focus to validate cellular systems to form spheroids, such as Microtissues (mold system), which can form 35 spheroids/well in multi-well plates (Svoronos et al. 2014). In addition, a future step could consist of performing a quantitative analysis of the dose response effect from chemicals to quantify the chemical potency and determine the point of departure (POD) through BMD analysis (Thienpont et al. 2024).

Altogether, this study demonstrates the applicability of TGx-DDI and GENOMARK transcriptomic biomarkers for classifying GTx, non-GTx, and NGTxC compounds in 3D HepaRG spheroids using gene expression data generated by the TempO-Seq sequencing technology. Both biomarkers displayed a high accuracy in the classification of the tested chemicals in a 3D HepaRG spheroid model, especially when evidence from both biomarkers was combined. Finally, this study highlights the potential of applying more physiologically relevant models, such as 3D HepaRG spheroids, to enhance genotoxicity assessment.

## Supplementary Material

kfag095_Supplementary_Data
