# Supplementary material for: Advancing genotoxicity assessment with transcriptomic biomarkers: from 2D to 3D HepaRG spheroid models
Source: Toxicol Sci. 2026 Aug 22;209(10):kfag095. doi: 10.1093/toxsci/kfag095 (PMC13641827; doi:10.1093/toxsci/kfag095)
Supplement: Supplementary file 1 [file kfag095_supplementary_data.docx]

**Supplementary Figure 1.** Pre-Cytotoxicity of the Genotoxic (GTx) selected chemical list (EMS, AFB1, BaP, CPA, and PA). The linear line represents the relative ATP percentage of the concentrations tested of each chemical with 3D HepaRG spheroids. Data presented as mean (three independent biological replicates) ± SEM.

**Supplementary Figure 2.** Pre-Cytotoxicity of the Non-genotoxic Non-carcinogens (non-GTxs) selected chemical list (CAFF, AMP, AFB2, 2DDG, and D-man). The linear line represents the relative ATP percentage of the concentrations tested of each chemical with 3D HepaRG spheroids. Data presented as mean (three independent biological replicates) ± SEM.

**Supplementary Figure 3.** Pre-Cytotoxicity of the Non-genotoxic carcinogens (NGTxC) selected chemical list (DEHP, TAA, DEA, PFOA, and TPA). The linear line represents the relative ATP percentage of the concentrations tested of each chemical with 3D HepaRG spheroids. Data presented as mean (three independent biological replicates) ± SEM.
